# Quick prioritization of Cochrane reviews on benign conditions of the prostate

**DOI:** 10.1002/cesm.12002

**Published:** 2023-03-27

**Authors:** Juan V. A. Franco, Jae H. Jung, Philipp Dahm

**Affiliations:** ^1^ Institute of General Practice Medical Faculty of the Heinrich‐Heine‐University Düsseldorf Düsseldorf Germany; ^2^ Department of Urology Yonsei University Wonju College of Medicine Wonju South Korea; ^3^ Centre of Evidence‐Based Medicine, Institute of Convergence Science Yonsei University Seoul South Korea; ^4^ Minneapolis VAMC, Urology Section and Department of Urology University of Minnesota Minneapolis Minnesota USA

**Keywords:** benign prostatic hyperplasia, Cochrane, prostatitis and prioritization/priority setting, systematic reviews

## Abstract

**Introduction:**

Benign conditions of the prostate include benign prostatic enlargement and prostatitis, which constitute an important cause of morbidity in men.

**Objective:**

We aimed to generate a list of priority topics of interest to our external stakeholders within our editorial scope.

**Methods:**

Following Cochrane's guidance, we developed a tiered approach for consulting internal and external stakeholders, including members from Urological societies. First, we analyzed our portfolio, including different impact measurements, to assess the need for updates. Then, following criteria related to the feasibility, novelty and relevance of prospective topics for evidence synthesis, we narrowed the list to a suite of titles for updates or new reviews.

**Results:**

Twelve editors provided initial feedback as to what the priorities were for updating existing reviews and for new reviews in our portfolio. The editors identified gaps in our portfolio, mainly covering new treatments for benign prostatic hyperplasia. Then we consulted external stakeholders obtaining 30 responses from 14 countries. These stakeholders provided additional information about the relative importance of existing topics and suggested new ones. We identified that many of the latter were already covered in our portfolio, highlighting gaps in their dissemination. Finally, we narrowed down four priority topics that the editorial group will take forward and two additional topics that might need other considerations before being commissioned.

**Conclusions:**

Following Cochrane's guidance on priority setting, we identified topics relevant to our editors and external stakeholders by analysing our portfolio and two rounds of surveys. Moreover, we identified opportunities for disseminating existing reviews. Further evaluation is needed of the following up commissioning process for priority reviews.

## 1 INTRODUCTION

Cochrane Urology is one of the over 50 editorial groups of Cochrane, an international not‐for‐profit organization that aims to promote the use of evidence in healthcare by producing high‐quality systematic reviews free from conflict of interest. This group currently holds a portfolio of protocols and reviews dedicated to the diagnosis, prevention, treatment, and rehabilitation of benign and malignant prostate conditions, male sexual dysfunction, benign and malignant renal conditions, and urologic cancers. The group is based at the University of Minneapolis and has a Korean Satellite at Yonsei University in Wonju. Cochrane Urology reviews are not only published in the *Cochrane Database of Systematic Reviews*, but they are also usually co‐published in specialty journals like the *BJUI International, Investigative and Clinical Urology* and *World Journal of Men's Health* for wider dissemination.

Since 2014, the group has not conducted a formal process for the prioritization of review topics. The editorial team relies on the submission of proposals by author teams and the input of the content expertise of the editors. Prioritizing topics for evidence synthesis can increase the relevancy of reviews, reduce research waste, and, by engaging with stakeholders, increase their uptake for decision‐making [1, 2, 3, 4].

One of the main topics covered by our group is diseases of the prostate. The prostate gland is an organ about the size of a walnut that lies below the urinary bladder that surrounds the male urethra. The prostate can be affected by malignant conditions (cancer) or benign conditions (inflammation or enlargement). Prostate cancer is the second most common cancer and the fifth leading cause of cancer death in men [5], and benign prostate conditions, especially benign prostate enlargement, have the highest burden of disease in men [6, 7]. According to the International Classification of Diseases 11 (ICD‐11), benign diseases of the prostate are classified into hyperplasia of the prostate (benign prostatic hyperplasia or benign prostatic enlargement) and inflammatory diseases, where the different forms of prostatitis stand out [8].

A recent systematic review identified a wide variety of steps to prioritize evidence synthesis, which can be grouped into three phases: a) a pre‐prioritization phase aimed at collecting data, planning and selection of topics, b) a prioritization phase aimed at analysing evidence gaps, establishing criteria and draw rankings, and c) a postprioritization phase aimed at effectively implementing the priorities, with subsequent monitoring and evaluation [9]. In this manuscript, we described the activities of our group aimed at generating a list of priority topics of interest to our stakeholders within our editorial scope and a special focus on benign conditions of the prostate. We report the findings of this process following the Reporting guideline for priority setting of health research (REPRISE) [10].

## 2 METHODS

### 2.1 Context and scope of prioritization

*Geographical scope*: Cochrane is a global organization; therefore, we aimed to provide evidence of global relevance. We sampled a wide variety of stakeholders for this purpose (see below).

*Health topic and intended beneficiaries*: Due to the broad scope of the Group's review portfolio and limited resources for conducting prioritization activities, the first part of this project focused on men with benign conditions of the prostate, including prostatitis and lower urinary tract symptoms (LUTS) due to benign prostatic hyperplasia (BPH). Previous research has highlighted that the number of Cochrane reviews underrepresented the burden of these conditions [11].

*Target audience of the priorities*: Our group focused on healthcare professionals providing care for men with benign conditions of the prostate with a focus on urologists and professional organizations developing clinical practice guidelines.

*Research area and type of research questions*: Clinical research with a focus on diagnostic, prognostic and intervention reviews.

### 2.2 Governance and team

Our steering group was composed of the Coordinating Editors from the (PD from the USA, JHJ from Korea), a contact editor and project lead (JVAF in Argentina/Germany) with support from the managing editors (Robert Lane and Jennifer Mariano in the USA). PD and JHJ are urologists and JVAF is a family physician. We received methodological support from the Cochrane's Editorial & Methods Department (see Acknowledgments).

### 2.3 Framework for priority setting

The full protocol for this process was published on Cochrane's websites and in Open Science Framework, and we followed the framework of Cochrane's Priority Setting Guidance Note [12, 13]. This Guidance outlines five different scenarios that Cochrane Review Groups may choose to follow with regard to priority setting depending on the breadth of the project, and the availability of resources. This project was aligned with the scenario of a “quick update and prioritization” based on our limited resources and included an analysis of our current portfolio and two rounds of feedback from editors and external stakeholders in the field of urology. This approach was chosen based on the previous experience of other Cochrane Groups [14, 15, 16, 17].

### 2.4 Stakeholders or participants

We aligned our inclusion criteria with our target audience of the priorities, but we also considered the wider participation of policymakers and patients, although our snowball sampling strategy was not aimed at these groups. We gathered contact information (email addresses) from three sources: a) A map of stakeholders provided by the Cochrane Cancer Network, which included patient organizations, funders and others (while the focus was on cancer, some stakeholders focused on problems of the prostate more broadly, including benign conditions), b) The list of 99 urological associations worldwide available at the American Urological Association website (https://www.auanet.org/education/global-academic-exchanges/international-societies/, last access October 2021, now not available), c) Personal contact through snowball sampling through our editors and mailing lists (mostly guideline developers). Our surveys (see below) were also disseminated through Twitter and our websites. We did not provide compensation for participation in this project, but we offered acknowledgment. Participants were asked to provide their email addresses if they wanted the final published report of the prioritization project. This project was approved by the institutional review board of Instituto Universitario Hospital Italiano de Buenos Aires (Approval number 0038‐2020).

### 2.5 Identification and collection of research priorities

*Collecting initial priorities*: We collated all reviews and protocols registered in our Cochrane Group within the scope of benign conditions of the prostate alongside metrics of impact (citations, Altmetrics scores, and citations in guidelines). Then we sent a survey to our editors, asking them to rate the topics’ importance for update and suggestions for new topics. After collating and summarizing the responses, we sent a second survey to the stakeholders described in the first section. In this survey we asked them to rate the importance of topics in our portfolio and those suggested by our editors. We also asked them to propose new topics. The surveys were created and distributed with SurveyMonkey (Momentive Inc.)

*Collating, categorizing and modifying priorities*: New topics following the PICO format were worded according to the guidance of the Cochrane Handbook for naming reviews and reviewed by the steering group. We deduplicated topics and mapped those that overlapped and we also identified questions in which larger or smaller reviews could be framed.

*Identifying overlapping reviews*: We consulted our editors and ran exploratory searches for non‐Cochrane systematic reviews indexed in MEDLINE in the last 5 years.

### 2.6 Prioritization of topics

We held an online editorial meeting where we presented the data summarized in the previous section, and we identified the main topics we prioritized for updates or the commission of new reviews by discussion and consensus. We created a PowerPoint presentation with the data available in Appendix, and we discussed the status of ongoing reviews and teams.

Our main criteria for prioritization included some of the FINER criteria for primary research [18] (*Feasible, Interesting, Novel, Ethical and Relevant*):

- **Feasibility**: considering the methodological challenges, the potential size of the review, the availability of primary studies and existing high‐quality systematic reviews on this topic.
- **Relevance and novelty**: for patients, clinicians and guideline developers, including areas of ongoing controversies or where new technologies, drugs or procedures are being implemented.

We did not include the criterion ‘interesting’ since we worked under the assumption that the priorities would be picked up by groups of review authors who would be highly interested in those topics. Conversely, small research groups could have strong interests in topics with little relevance for practice. Moreover, the criteria ‘Ethical’ from this framework would not be applicable for systematic reviews.

Based on these criteria, the priorities of our editors and external stakeholders were consolidated. We did not use a formal ranking system at this stage, reaching consensus was the priority, and no areas of conflict or dissent arose during this process; however, if present, we had planned it to be handled by our Co‐ordinating editor. We did not seek further feedback from external stakeholders at this stage.

## 3 RESULTS

### 3.1 Results from the surveys

For the first round of surveys, 12 out of 14 editors replied (80% response rate). The remaining two editors because their methodological or clinical expertise fell outside the scope of this project. The topics that they prioritized for update or completion were:

- Antimicrobial therapy for chronic bacterial prostatitis—review from 2013 (67%).
- Anticholinergics combined with alpha‐blockers for LUTS—protocol from 2016 (50%).
- Microwave thermotherapy for LUTS—review from 2012 (42%).
- 5‐alpha‐reductase inhibitors for LUTS due to BPH—protocol from 2015 (42%).
- *Serenoa repens* for BPH—review from 2012 (25%).
- *Pygeum africanum* for BPH—review from 1998 (17%).
- Beta‐sitosterols for BPH—review from 1999 (8%).
- Desmopressin for treating nocturia in men—protocol from 2016 (8%).

The editors provided feedback as to how the review questions could be revised (merging phytotherapy or focusing on combination therapy for 5‐ARI). Moreover, two of the top‐rated topics (anticholinergics had already been identified for prioritization and the publication was scheduled in 2021 [19, 20]. The editors also suggested the following new topics:

- β3 adrenoceptor agonists (Mirabegron) for lower urinary tract symptoms (LUTS) due to benign prostatic hyperplasia (BPH).
- High‐intensity focused ultrasound (HIFU) for LUTS due to BPH.
- Urodynamic studies for the management of BPH.
- Photovaporisation for the treatment of BPH.
- Thulium enucleation for the treatment of BPH.
- Prognostic factors for the progression of LUTS.
- Diagnostic test accuracy of investigations for bladder outlet obstruction in men.

**Figure 1:**
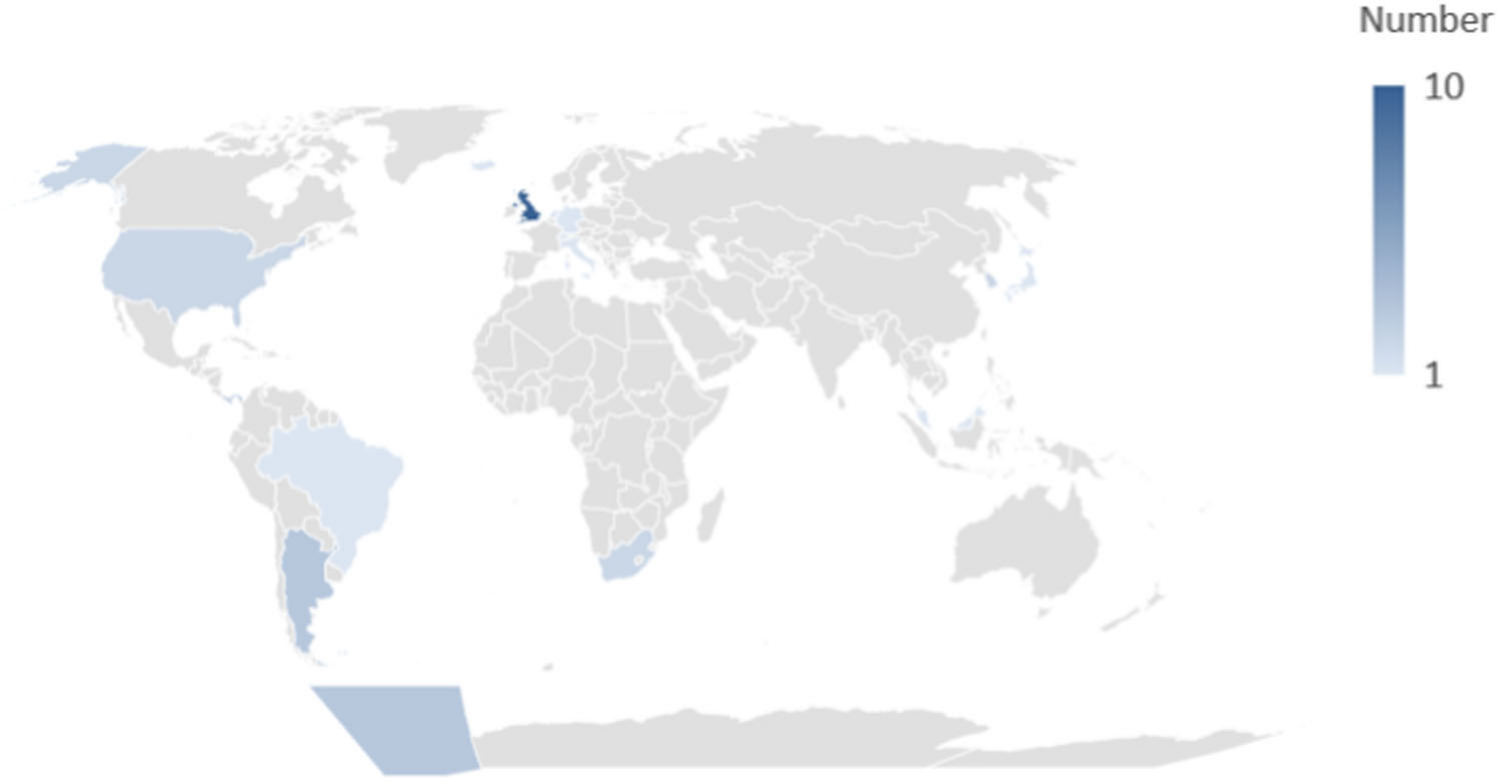
Geographical distribution of respondents (blue‐gradient scale). Argentina 3, Brazil 1, Germany 1, Iceland 1, Italy 1, Japan 1, Malaysia 1, Netherlands 1, Panama 3, South Africa 2, South Korea 2, Switzerland 1, United Kingdom 10, United States 2.

For our second round of the survey, we received 30 responses from external stakeholders, of which 27 (90%) identified as physicians, seven as researchers, six as members of a scientific organization, five as guideline developers, three as carers of someone affected by prostatic diseases, four as systematic reviewers, two as policymakers, and one as someone affected by a prostatic disease. We cannot calculate a precise response rate due to the sampling method (snowball). The geographical distribution was diverse (see Figure 1).

The topics prioritized for update by these stakeholders were:

- Antimicrobial therapy for chronic bacterial prostatitis (70%).
- 5‐alpha‐reductase inhibitors for LUTS due to BPH (50%).
- Desmopressin for treating nocturia in men (33%).
- *Serenoa repens* for BPH (27%).
- *Pygeum africanum* for BPH (7%).
- Beta‐sitosterols for benign prostatic hyperplasia (0%).

Many external stakeholders listed new review topics already covered in our portfolio, which may indicate a need to disseminate our work further. Nonetheless, removing these overlapping topics, we identified the following new topics:

- Surgical management comparison
  ○ Efficacy of robotic simple prostatectomy compared to HoLEP.
  ○ Robot simple prostatectomy for the treatment of lower urinary tract symptoms in men with benign prostatic hyperplasia.
  ○ Photoselective vaporization (PVP).
  ○ Anatomical endoscopic enucleation of the prostate (AEEP).
- Other medical therapies for LUTS:
  ○ Early surgery (relative indications) versus medical treatment of BPH.
  ○ Doxazosin for treatment of lower tract symptoms in men with BPH.
  ○ Use of *Serenoa repens* for the treatment of Luts in men with dysmetabolic diseases.
  ○ B3 receptor agonist for the treatment in men with BPH.
  ○ Role of Imipramine in the treatment of severe voiding symptoms.
- Diagnosis and LUTS:
  ○ Accuracy of digital rectal examination in the diagnosis of benign prostatic hyperplasia in general practitioners.
  ○ Accuracy of cut‐off of uroflow for detecting obstruction.
  ○ Accuracy of prostate ultrasonography in the diagnosis of benign prostatic hyperplasia/Role of digital rectal examination.

Finally, external stakeholders rated the importance of new topics suggested by our editors (in brackets weighted average, range 1–5, ordered by decreasing importance):

- Prognostic factors for the progression of LUTS (4.33).
- Diagnostic test accuracy of investigations for bladder outlet obstruction in men (4.27).
- Urodynamic studies for the management of BPH (3.73).
- β3 adrenoceptor agonists (Mirabegron) for lower urinary tract symptoms (LUTS) due to benign prostatic hyperplasia (BPH) (3.67).
- Thulium enucleation for the treatment of BPH (3.37).
- Photo vaporization for the treatment of BPH (3.1).
- High‐intensity focused ultrasound (HIFU) for LUTS due to BPH (2.67).

### 3.2 Final editorial prioritization

Considering the input from our editors and external stakeholders, we held an editorial meeting in February 2022. We identified two topics that needed updating and two new topics that may be covered in Cochrane reviews:

- 5‐alpha‐reductase inhibitors for lower urinary tract symptoms secondary to benign prostatic obstruction (update of the 2018 review).
- *Serenoa repens* for the treatment of LUTS (update of the 2012 review).
- Robotic simple prostatectomy for LUTS (new review).
- Early procedures vs medical treatment of BPH (new review).

Additionally, going through the list of topics, we identified that there is an opportunity to invite authors that produce high‐quality systematic reviews outside of Cochrane to create new and up‐to‐date Cochrane reviews on the following topics, but there were some additional considerations:

- Diagnostic test accuracy of investigations for bladder outlet obstruction in men/Prognostic factors for the progression of lower urinary tract symptoms: these reviews require special expertise in Cochrane reviews on diagnostic and prognosis.
- β3 adrenoceptor agonists (Mirabegron) for lower urinary tract symptoms due to benign prostatic hyperplasia: this review overlaps with the scope of the Cochrane Incontinence Group (which covers overactive bladder syndrome).

Unlike other topics in which we identified a clear PICO question, the focus on the diagnostic accuracy of investigations and prognostic factors for the progression of symptoms was not well defined. The type of diagnostic investigations may include a wide array of possibilities, including digital rectal examination and prostatic ultrasonography. Moreover, some prognostic factors, including age, are well known to predict the onset and progression of symptoms, so it would be important to know what is the knowledge gap that needs to be covered.

## 4 DISCUSSION

### 4.1 Postprioritization: Monitoring, evaluation, and feedback

Of the four prioritized topics, two are either advanced in their development (5‐alpha‐reductase inhibitors for lower urinary tract symptoms secondary to benign prostatic obstruction) or have already been submitted for publication (*Serenoa repens* for the treatment of lower urinary tract symptoms). Our CRG is yet to define the scope and coverage of the other two new reviews, considering that the evidence from RCTs might be lacking and observational studies might shape the reviews (robotic simple prostatectomy for LUTS) or the evidence base depends on ongoing studies (early procedures vs medical treatment of BPH). For the former, a scoping review of existing evidence may better inform the conduct of subsequent reviews. For the latter, our group receives weekly updates from Pubmed/MEDLINE to survey published trials. Our CRG will also assess funding opportunities and the possibility of recruiting and providing support to new authors to increase capacity in systematic review development.

Our project suffered several delays due to the lack of specific funding and a low response rate from external stakeholders. We intended to repeat this project 6 months after its completion within another topic of our scope, but we have been unable to do so due to a lack of capacity. We also decided not to use ranking or scoring in all stages of the process, which could have resulted in a quantitative output for prioritization since we believed that meaningful discussions from our editorial board would be more productive in prioritizing and re‐shaping review questions. We found this was a preferred approach even in larger and funded priority‐setting exercises such as those conducted by the Cochrane Consumers and Communications Group [21]. Additionally, the prioritization criteria (feasibility, relevance and novelty) could have been defined alternatively and heavily relied on subjective judgements. For instance, feasibility may depend on access to funding or greater collaboration; however, certain aspects, such as the availability of ongoing or published primary studies, are key when defining priorities. We are confident in our assessment of the relevance, considering the history of the group and its international board, which frequently engages key stakeholders, including guideline developers. Novelty may also have important equity considerations since the availability of technology and drugs for benign conditions of the prostate vary widely across settings. Our low response rate limits the representativity of the views and perspectives of external stakeholders. For instance, we contacted 14 Asian associations of urology, where there is a high incidence of this condition, but we received few responses, mostly from South Korea, where one of the editorial bases of the Group sits. This highlights the need to develop and grow meaningful and engaging links with stakeholders before consultation.

External stakeholders highlighted the need for reviews on minimally invasive treatments and desmopressin for lower urinary tract symptoms, for which there is a suite of Cochrane reviews [20, 22, 23, 24, 25, 26]. The CRG strategy for dissemination was co‐publication of those reviews [22, 27, 28, 29], which has been shown to improve the impact of Cochrane reviews by reaching a highly specialized readership [30]. Moreover, both reviews have been cited in clinical practice guidelines. Nonetheless, additional actions may be taken to increase the visibility of our reviews. The Cochrane Framework for Knowledge Translation describes many activities beyond prioritization, including the engagement of stakeholders, push‐and‐pull strategies and translations, some of which cover the work of our Review Group [31]. The Group has ongoing communications with the main guideline developers from the American Urological Association and the European Society of Urology. Moreover, we engaged in small‐scale projects to package and push our reviews, including the Cochrane‐Wikipedia Initiative. Finally, Cochrane has partners across their geographic centers that provide translations of our reviews in multiple languages.

Finally, considering that a swift publication of reviews has not followed other prioritization projects, we will also have to monitor the production and editorial processing of priority topics [32]. Considering that the reviews from our Cochrane Review Group are above the Cochrane average in their use in guidelines, we will also have to monitor the uptake and feedback by our key stakeholders [33].

## 5 CONCLUSIONS

Through an analysis of our portfolio and two rounds of internal and external feedback, we identified high‐priority topics for our key stakeholders, including clinicians and guidelines developers. Additional evaluations of the impact of this process is needed, including the output of prioritized reviews and their use in clinical practice guidelines.

## AUTHOR CONTRIBUTIONS

**Juan V. A. Franco**: Conceptualisation, data curation, formal analysis, investigation, methodology, project administration. **Jae H. Jung**: Conceptualisation, formal analysis, investigation. **Philipp Dahm**: Conceptualisation, formal analysis, investigation.

## CONFLICT OF INTEREST STATEMENT

Philipp Dahm and Jae Hung Jung are Co‐ordinating editors, and Juan Franco is the contact editor for the Cochrane Urology Group. Juan Franco is also Managing Editor for the Cochrane Metabolic and Endocrine Disorders Group, elected member of Cochrane's Governing Board and Editor‐in‐Chief of BMJ Evidence‐Based Medicine, and Clinical Editor of the BMJ.

## ETHICS STATEMENT

This project was approved by the institutional review board of Instituto Universitario Hospital Italiano de Buenos Aires (Approval number 0038‐2020).

## Supporting information

Supporting information.

## Data Availability

All anonymized survey data is available upon request.
