# Supplementary material for: Quick prioritization of Cochrane reviews on benign conditions of the prostate
Source: Cochrane Evid Synth Methods. 2023 Mar 27;1(2):e12002. doi: 10.1002/cesm.12002 (PMC11795903; doi:10.1002/cesm.12002)
Supplement: Supplementary file 1 — Supporting information. [file CESM-1-e12002-s001.docx]

Priority setting process for Cochrane Urology

Focus: benign conditions of the prostate

Final report

Contents

[**Responses by the editors**](#_heading=h.iyx69a2hejpu) **2**

[Q1: Which topic do you consider that needs to be completed or updated most urgently?](#_heading=h.30j0zll) 3

[Q2. Which new topics within the scope of benign conditions of the prostate need to be addressed in a Cochrane review?](#_heading=h.1fob9te) 3

[Q3. Do you have any suggestions for how we could improve this priority-setting process?](#_heading=h.2et92p0) 4

[Q4. Is there any relevant stakeholder you would like us to invite for the second stage?](#_heading=h.tyjcwt) 5

[Q5. Any other comments:](#_heading=h.3dy6vkm) 5

[Q6. Responders from the editorial team (editors with clinical expertise):](#_heading=h.1t3h5sf) 5

[**Responses by external stakeholders**](#_heading=h.byxr0ubhgtkp) **8**

[Q1. Personal information](#_heading=h.qudyoljemnhj) 8

[Q2. Which role best describes you?](#_heading=h.v4v8p3qmtagk) 9

[Q3. Our current portfolio has the following outdated topics (defined as those published in a review or protocol before 2018). Which topic do you consider that needs to be completed or updated most urgently?](#_heading=h.gvwfus8zc9fl) 10

[Q4. These are the topics we are currently covering in our reviews and protocols.[...] Which new topics within the scope of benign conditions of the prostate need to be addressed in a Cochrane review? Indicate the rationale](#_heading=h.cnlfjhjom4e) 10

[Q5. Our editors have proposed that the following topics need to be updated or included in our portfolio of reviews. Could you please rate their importance?](#_heading=h.7pge9a25lrc5) 11

[Q6. Do you have any suggestions for how we could improve this priority setting process?](#_heading=h.1zh5a3918jt2) 12

[**Editorial consensus**](#_heading=h.lxc7o9vulrmz) **13**

[Editorial meeting on February 21st 2022](#_heading=h.8lhc3j3ypyyw) 13

[Priority topics for further exploration](#_heading=h.sbcr20c03uno) 13

# Responses by the editors

## Q1: Which topic do you consider that needs to be completed or updated most urgently?


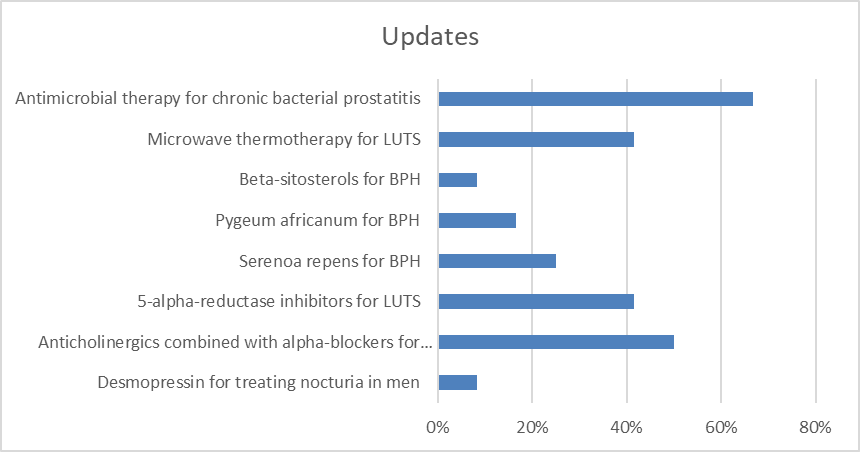


Comments on this question:

- “Could merge (herbal) BPH topics under one phototherapy banner.”
- “Just FYI, Anticholinergics review will be published soon if Philipp sign off on it. For 5ARI review, Andrew took over it from the original author team this year. In addition, I have updated search for Desmopressin review, but found only 3 RCTs that does not have any additional information.”
- “Maybe instead of *Serenoa repens*, we can include phytotherapy in general. 5ARI would ideally evaluate the effect of combination therapy.”
- “I will suggest that the evidence should be explored based upon when the study was conducted due to increased antibiotic resistance over the years.” [Note: I think this refers to antibiotic therapy for prostatitis]

**Suggestions after the input of the steering group:**

We should remove anticholinergics and transurethral microwave thermotherapy since they are advanced in the editorial process. Desmopressin and 5ARI should remain until the status update.

## Q2. Which new topics within the scope of benign conditions of the prostate need to be addressed in a Cochrane review?

| **Topic** | **Rationale** |
| --- | --- |
| In men with BPH, what is the impact of HIFU on LUTS | HIFU is approved for prostatic “ablation” and is increasingly used |
| Use of beta agonists for management of LUTS (Mirabegron, vibegron) | None provided |
| Prostatic artery embolization for treatment of benign prostate hyperplasia |  |
| Mirabegron for treatment of benign prostate hyperplasia |  |
| HIFU outcomes in prostate cancer |  |
| Mitogel outcomes for upper tract urothelial cancer |  |
| Overactive bladder |  |
| Peyronies |  |
| Erectile Dysfunction |  |
| The network meta-analysis on surgery for BPH- this is timely and much needed with the current expansion of new surgical modalities |  |
| Urodynamics and success of surgical treatment |  |
| Radiofrequency treatment | Lack of high-grade review |
| Nonpharmacological treatment for prostatitis |  |
| Mirabegrone (beta 3 agonists) for the treatment of lower urinary tract symptoms in men with benign prostatic hyperplasia | Beta 3 agonist is the latest medicine for BPH. |
| Surgical management – photo vaporisation |  |
| Surgical management - thulium enucleation |  |
| The role of urodynamic studies in BPE | Lots of resources into this, possibly little impact |
| Prognostic factors for the progression of LUTS |  |
| β3 adrenoceptor agonists (mirabegron) | newer treatment |
| PDE5 inhibitor for BPS |  |
| Diagnostic test accuracy of investigations for bladder outlet obstruction in males. |  |

**Suggestions after the input of the steering group:**

Some editors did not adequately understand this question since they introduced cancer topics. Maybe it would help if we do not display the whole portfolio and emphasise benign conditions of the prostate, and then we can weed out the topics already covered by our portfolio. Removing the topics covered by reviews after 2018 and those outside the scope of benign conditions of the prostate, some emerging topics for benign conditions of the prostate include:

- β3 adrenoceptor agonists (Mirabegron) for lower urinary tract symptoms (LUTS) due to benign prostatic hyperplasia (BPH)
- High-intensity focused ultrasound (HIFU) for LUTS due to BPH
- Urodynamic studies for the management of BPH
- Photo vaporisation for the treatment of BPH
- Thulium enucleation for the treatment of BPH
- Prognostic factors for the progression of LUTS
- Diagnostic test accuracy of investigations for bladder outlet obstruction in men.

## Q3. Do you have any suggestions for how we could improve this priority-setting process?

- “Great idea to put out this survey!”
- “I don’t think we need the diagnosis codes.”
- “Sharing project process using Google calendar or Dropbox. Send an alert email to the review team per month.”
- “Inclusion of experienced review authors.”

**Suggestions after the input of the steering group:**

We should remove the diagnostic codes. The other suggestions relate to review production.

## Q4. Is there any relevant stakeholder you would like us to invite for the second stage?

- “AUA, EAU, DGU (German society), Korean Society of Urology”
- “Guideline Panels”
- “Latin American Associations of Urology”

**Suggestions after the input of the steering group:**

We should include these stakeholders in round 2 (some were already identified).

## Q5. Any other comments:

- “Really appreciate the Altimetric scores!”
- “It would be worth investigating whether Cochrane could perform systematic reviews for certain guideline groups and in exchange, co-publication can be in the respective journal.”

**Suggestions after the input of the steering group:**

Co-publication will be explored in the near future.

## Q6. Responders from the editorial team (editors with clinical expertise):

Vikram Narayan, Philipp Dahm, Eu Chang Hwang, Giulia Lane, Niranjan, Michael Risk, Kourosh Afshar, Jae Hung Jung, Andrew Shepherd, Juan Franco, Frank Kunath, Imran Omar

# Sources for external stakeholders

### Urological associations and date of contact in 2021 (N/A indicates that no email was available)

South America

- Sociedad Argentina de Urología 15/09
- Confederacion Americana de Urologia 15/09
- Federación Argentina de Urología 15/09
- Sociedad Boliviana de Urología 15/09
- Sociedad Brasileira de Urología 15/09
- Sociedad Chilena de Urología 15/09
- Sociedad Colombiana de Urología 15/09
- Sociedad Ecuatoriana de Urología 15/09
- Sociedad Peruana de Urología 15/09
- Sociedad Uruguaya de Urología 15/09
- Sociedad Venezolana de Urología 15/09

North America

- Canadian Urological Association 22/09
- Caribbean Urological Association 22/09
- Colegio Mexicano de Urología Nacional, A.C. 22/09
- Jamaica Urological Society 22/09
- Stan Alger (AUA) 22/09
- Aurelie M. Alger (AUA) 22/09
- Michele Paoli (AUA) 22/09
- Ms Wendy Weiser (AUA) 22/09
- Drew Shifflet (AUA) 22/09
- Puerto Rico Urology Association 22/09
- Sociedad Cubana de Urologia 22/09
- Sociedad Dominicana de Urología 22/09
- Sociedad Mexicana de Urologia 22/09
- Societe Internationale D'Urologie 22/09
- Ms Wendy Weiser (AUA) 22/09
- Ms Wendy Weiser (AUA) 22/09
- Frank J. DeSantis (AUA) 22/09
- Asociación de Médicos Urólogos de Costa Rica 22/09
- Asociaciòn Guatemalteca de Urología 22/09
- Asociación Urología de Nicaragua N/A
- Sociedad Hondureña de Urologia 22/09
- Sociedad Panameña de Urología 22/09
- Asociación Salvadoreña de Urología 22/09

Asia

- Korean Urological Association 25/08
- Bangladesh Association of Urological Surgeons 25/08
- Chinese Urological Association 25/08
- Hong Kong Urological Association 08/09
- Indonesian Urological Association 08/09
- Japanese Urological Association 08/09
- Malaysian Urological Association 08/09
- Pakistan Association of Urological Surgeons 08/09
- Philippine Urological Association N/A
- Singapore Urological Association 08/09
- Sri Lanka Association of Urological Surgeons 08/09
- Taiwan Urological Association 08/09
- Thai Urological Association 08/09
- Urological Association of Asia 08/09
- Urological Society of India 08/09
- Vietnam Urological Association N/A

Middle East

- Saudi Urological Association 25/08
- Lebanese Urological Society 25/08
- Israeli Urological Association 25/08
- Emirates Urological Society 25/08
- Iranian Urological Association 25/08
- Arab Association of Urology 25/08

Europe

- Albanian Society of Urology N/A
- Asociación Española de Urología 08/09
- Associação Portuguesa de Urologia 08/09
- Association Francaise d'Urologie 08/09
- Associacao Lusofona De Urologia N/A
- Austrian Association of Urology 08/09
- Belgian Association of Urology 08/09
- British Association of Urological Surgeons 08/09
- Croatian Urological Association 08/09
- Czech Urological Society 08/09
- Danish Urological Society 08/09
- Deutsche Gesellschaft für Urologie 08/09
- Estonian Urological Society 08/09
- EAU 08/09
- Finnish Urological Association 08/09
- Georgian Association of Urology 08/09
- Hellenic Urological Association 08/09
- Hungarian Society of Urology 08/09
- Iceland Urological Association 08/09
- Irish Society of Urology 08/09
- Latvian Urological Association 15/09
- Lithuanian Urology Society 15/09
- Northern Macedonian Society of Urology N/A
- Nederlandse Vereniging Voor Urologie 15/09
- Norwegian Urological Association N/A
- Polish Urological Association 15/09
- Romanian Association of Urology 15/09
- Russian Society of Urology 15/09
- Scandinavian Association of Urology 15/09
- Slovak Association of Urology 15/09
- Slovenian Urological Association 15/09
- Societe Belge d'Urologie 15/09
- Societá Italiana di Urologia 15/09
- Schweizerische Gesellschaft fur Urologie 15/09
- Society of Urological Surgery in Turkey 15/09
- Swedish Association of Urology 15/09
- Turkish Association of Urology 15/09
- Urological Association of Serbia 15/09

Africa

- Egyptian Urological Association 25/08
- Tunisian Urological Society 25/08
- Pan African Urological Surgeons Association 25/08
- South African Urological Association 25/08

Australia

- ASANZ 25/08

### Social media

Twitter accounts that tweeted the poll: @C_Incontinence, @cochranenutri, @cochranecollab, @CCGlobalAgeing, @CochraneUK, @CochranePaPaS, @CochraneLibrary, @CochraneUrology, @EBMUrology, @juan_francomd

### Websites and partners

- Fields and Groups: Incontinence CRG and Global Ageing Field
- Cochrane's Mailing List
- Cochrane's Website: https://community.cochrane.org/news/current-cochrane-group-priority-setting-projects
- Cochrane Urology Website: <https://urology.cochrane.org/>

### Key opinion leaders and stakeholders (data not available for sharing due to data protection)

# Responses by external stakeholders

## Q1. Personal information

| **Answer Choices** | **Responses** | |
| --- | --- | --- |
| Allied-health professional (e.g. pharmacist, dietician, physiotherapist, etc.) | 0.00% | 0 |
| Carer of someone who is affected by a prostatic disease | 10.00% | 3 |
| Guideline developer | 16.67% | 5 |
| Member of a scientific association | 20.00% | 6 |
| Nurse | 0.00% | 0 |
| **Physician** | **90.00%** | **27** |
| Policymaker | 6.67% | 2 |
| Researcher | 23.33% | 7 |
| Research funder | 0.00% | 0 |
| Someone who is or has been affected by a prostatic disease | 3.33% | 1 |
| Systematic reviewer (Cochrane or non-Cochrane) | 13.33% | 4 |
| Work at an advocacy organisation | 0.00% | 0 |
| Other | 3.33% | 1 |
|  | Answered | 30 |

**Location**


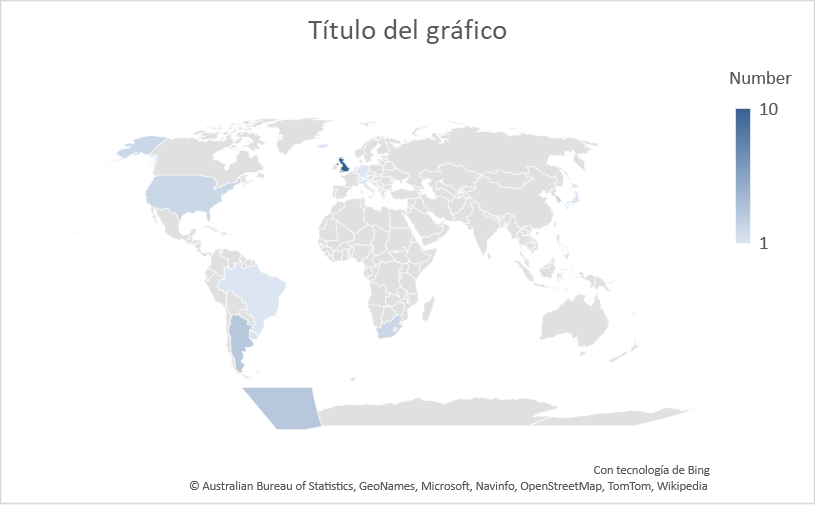


**Footnote:** Argentina 3, Brazil 1, Germany 1, Iceland 1, Italy 1, Japan 1, Malaysia 1, Netherlands 1, Panama 3, South Africa 2, South Korea 2, Switzerland 1, United Kingdom 10, United States 2

Researchers: 2 UK, 1 Netherlands, 1 USA, 1 Switzerland, 1 Japan, 1 South Korea

Physician: 8 UK, 1 Netherlands, 1 USA, 1 Brasil, 3 Panama, 1 Iceland, 1 Malaysia, 2 South Africa, 1 Italy, 1 Germany, 1 Switzerland, 2 South Korea, 1 Japan, 3 Argentina

Policymaker: 1 UK, 1 Switzerland

Systematic reviewer: 1 Netherlands, 1 USA, 1 Italy, 1 South Korea

Member of scientific organisation: 1 UK, 1 Netherlands, 1 USA, 1 Malaysia, 1 Italy, 1 South Korea

Someone affected by prostatic disease: 1 USA

Carer of someone affected by prostatic disease: 1 UK, 1 Malaysia, 1 South Korea

Guideline developer: 2 USA, 1 Italy, 1 Switzerland, 1 South Korea

## Q2. Which role best describes you?

| **Answer Choices** | **Responses** | |
| --- | --- | --- |
| Optional: Email address (if you want to receive the final report of this project) | 60.00% | 18 |
| Optional: Name (if you want to be publicly acknowledged, your individual responses will be confidential) | 46.67% | 14 |
| Affiliation (if you are not affiliated to an institution, e.g. AUA, University, etc., please type ‘none’)  ABUHB  *BAUS 3, King's College Hospital, BAUS/AUA, Jeroen Bosch Ziekenhuis, Albany Medical College, Royal Berkshire Hospital,*  *AUA, AUA/EAU/SIU/SPU/SRS, EAU, Hospital Universiti Sains Malaysia, SAUA, University Hospital Freiburg, Hanover Medical School, Yonsei University, Kyoto University, AAMF, Hospital Italiano de Buenos Aires 2, Chonnam National University Medical School* | 83.33% | 25 |

## Q3. Our current portfolio has the following outdated topics (defined as those published in a review or protocol before 2018). Which topic do you consider that needs to be completed or updated most urgently?

| Answer Choices | Responses | |
| --- | --- | --- |
| Desmopressin for treating nocturia in men (review 2017) | 33.33% | 10 |
| **5-alpha-reductase inhibitors for lower urinary tract symptoms secondary to benign prostatic obstruction (protocol 2015)** | 50.00% | 15 |
| Serenoa repens for benign prostatic hyperplasia (review 2012) [this could be updated covering phytotherapy] | 26.67% | 8 |
| Pygeum africanum for benign prostatic hyperplasia (review 1998) | 6.67% | 2 |
| Beta-sitosterols for benign prostatic hyperplasia (review 1999) | 0.00% | 0 |
| **Antimicrobial therapy for chronic bacterial prostatitis (review 2013)** | 70.00% | 21 |
| Any comments on these topics? |  | 3 |
|  | Answered | 30 |

## Q4. These are the topics we are currently covering in our reviews and protocols.[...] Which new topics within the scope of benign conditions of the prostate need to be addressed in a Cochrane review? Indicate the rationale

1. Prostatitis/Chronic prostatic pain syndrome/ difficult topic to advise
   1. Pelvic floor therapy for chronic pelvic pain in men (already covered)
2. Surgical management comparison
   1. Efficacy of robotic simple prostatectomy compared to HoLEP
   2. Robot simple prostatectomy for the treatment of lower urinary tract symptoms in men with benign prostatic hyperplasia
   3. Urolift / Rezum for the treatment of retention and success in terms of catheter free rates / PAE / Aquablation (already covered)
   4. Bipolar enucleation of the prostate / a lot of evidence available (reviews in progress)
   5. Comparison in the clinical outcome of TURP and MIST in men with BPH / The relative indications/effectiveness of LUTS surgical options when compared with each other / Retreatment rates and types after BPH surgery / Cost comparison of TURP and MIST in men with BPH (already covered)
   6. Photoselective vaporisation (PVP)
   7. Anatomical endoscopic enucleation of the prostate (AEEP)
3. Nocturia (already covered)
   1. Desmopressin for Men with nocturia - clinical effectiveness
   2. Desmopressin. Under utilisation currently. Needs robust dosage and management plans in the elderly with cardiac and renal disease.
   3. Desmopressin for Men with nocturia - safety Desmopressin for Men with nocturia - the need for urea and electrolyte monitoring
4. Other medical therapies for LUTS:
   1. Early surgery (relative indications) vs medical treatment of BPH
   2. Doxazosin for treatment of lower tract symptoms in men with BPH
   3. MIRABEGRON for the treatment of Urge incontinence post TURP (Overlap Cochrane Incontinence Group)
   4. Use of Serenoa repens for the treatment of Luts in men with dysmetabolic diseases
   5. B3 receptor agonist for the treatment in men with BPH
   6. Silodosin for male LUTS (already covered)
   7. Role of Imipramine in the treatment of severe voiding symptoms
5. Diagnosis and LUTS:
   1. Accuracy of digital rectal examination in the diagnosis of benign prostatic hyperplasia in general practitioners.
   2. Accuracy of cut-off of uroflow for detecting obstruction
   3. Accuracy of prostate ultrasonography in the diagnosis of benign prostatic hyperplasia / Are men in which digital rectal examination not used for the diagnosis of benign prostatic hyperplasia at higher risk of adverse health outcomes, including prostate cancer? (In other words, can the digital rectal examination be safely omitted?)
6. Beyond scope of this prioritisation project: Vitaros (alprostadil) - efficacy in Male ED, Varicoceles, Andrology, Expulsive treatment for ureterolithiasis, First line therapy for mRCC, Screening, Sexual Activity and multiple vs single partner. Heterosexual and Homosexual couples.

## Q5. Our editors have proposed that the following topics need to be updated or included in our portfolio of reviews. Could you please rate their importance?

|  | Not important at all | Low importance | Neutral | Important | Very important | Weighted Average |
| --- | --- | --- | --- | --- | --- | --- |
| β3 adrenoceptor agonists (Mirabegron) for lower urinary tract symptoms (LUTS) due to benign prostatic hyperplasia (BPH) | 0.00% | 16.67% | 20.00% | 43.33% | 20.00% | 3.67 |
| High-intensity focused ultrasound (HIFU) for LUTS due to BPH | 6.67% | 46.67% | 23.33% | 20.00% | 3.33% | 2.67 |
| Urodynamic studies for the management of BPH | 0.00% | 10.00% | 20.00% | 56.67% | 13.33% | 3.73 |
| Photo vaporisation for the treatment of BPH | 3.33% | 26.67% | 33.33% | 30.00% | 6.67% | 3.1 |
| Thulium enucleation for the treatment of BPH | 3.33% | 13.33% | 36.67% | 36.67% | 10.00% | 3.37 |
| Prognostic factors for the progression of LUTS | 0.00% | 0.00% | 6.67% | 60.00% | 33.33% | 4.27 |
| Diagnostic test accuracy of investigations for bladder outlet obstruction in men | 3.33% | 0.00% | 10.00% | 33.33% | 53.33% | 4.33 |

## Q6. Do you have any suggestions for how we could improve this priority setting process?

- Consult with opinion leaders in the field on an ongoing basis to assist with final choices
- This survey is fine.
- The members of systematic review must be well organised

# Editorial consensus

## Editorial meeting on February 21st 2022

There are some topics for which there is evidence but the results of the review would not be relevant to current practice:

- Thulium enucleation for the treatment of lower urinary tract symptoms
- Antimicrobial therapy for chronic bacterial prostatitis
- Urodynamic studies for the management of lower urinary tract symptoms
- Photoselective vaporisation
- Anatomical endoscopic enucleation of the prostate
- Doxazosin for treatment of lower tract symptoms in men
- Role of Imipramine in the treatment of severe voiding symptoms
- Accuracy of digital rectal examination in the diagnosis of benign prostatic hyperplasia
- Accuracy of cut-off of uroflow for detecting obstruction
- Accuracy of prostate ultrasonography in the diagnosis of benign prostatic hyperplasia

### Priority topics for further exploration

There is an opportunity to invite authors that produce high-quality systematic reviews outside of Cochrane to create new and up-to-date Cochrane reviews:

- Diagnostic test accuracy of investigations for bladder outlet obstruction in men
- Prognostic factors for the progression of LUTS
- β3 adrenoceptor agonists (Mirabegron) for lower urinary tract symptoms (LUTS) due to benign prostatic hyperplasia (BPH)

There are some topics that would need an update or a new review:

- 5-alpha-reductase inhibitors for lower urinary tract symptoms secondary to benign prostatic obstruction (update)
- Use of Serenoa repens for the treatment of LUTS (update)
- Robotic simple prostatectomy for LUTS (new review)
- Early procedures vs medical treatment of BPH (new review)
